# Repeated hapten exposure induces persistent tactile sensitivity in mice modeling localized provoked vulvodynia

**DOI:** 10.1371/journal.pone.0169672

**Published:** 2017-02-03

**Authors:** Jasmine Landry, Tijana Martinov, Hanna Mengistu, Jyothi Dhanwada, Charles J. Benck, Jaclyn Kline, Beebie Boo, Linnea Swanson, Elena Tonc, Randy Daughters, Brian T. Fife, Devavani Chatterjea

**Affiliations:** 1 Biology Department, Macalester College, St. Paul, Minnesota, United States of America; 2 Center for Immunology, University of Minnesota, Minneapolis, Minnesota, United States of America; University of Kansas Medical Center, UNITED STATES

## Abstract

**Background:**

Vulvodynia is a remarkably prevalent chronic pain condition of unknown etiology. Epidemiologic studies associate the risk of vulvodynia with a history of atopic disease. We used an established model of hapten-driven contact hypersensitivity to investigate the underlying mechanisms of allergy-provoked prolonged sensitivity to pressure.

**Methods:**

We sensitized female ND4 Swiss mice to the hapten oxazolone on their flanks, and subsequently challenged them four days later with oxazolone or vehicle for ten consecutive days on the labia. We evaluated labiar sensitivity to touch, local mast cell accumulation, and hyperinnervation after ten challenges.

**Results:**

Oxazolone-challenged mice developed significant tactile sensitivity that persisted for over three weeks after labiar allergen exposures ceased. Allergic sites were characterized by mast cell accumulation, sensory hyper-innervation and infiltration of regulatory CD4^+^CD25^+^FoxP3^+^ T cells as well as localized early increases in transcripts encoding Nerve Growth Factor and nerve-mast cell synapse marker Cell Adhesion Molecule 1. Local depletion of mast cells by intra-labiar administration of secretagogue compound 48/80 led to a reduction in both nerve density and tactile sensitivity.

**Conclusions:**

Mast cells regulate allergy-provoked persistent sensitivity to touch. Mast cell-targeted therapeutic strategies may provide novel means to manage and limit chronic pain conditions associated with atopic disease.

## Introduction

Vulvodynia is chronic vulvar pain of unknown etiology, diagnosed in the absence of obvious infections or overt inflammation [1, 2]. As many as 8% of women in the United States are likely to experience symptoms consistent with vulvodynia by the age of 40 [3] most presenting with provoked, localized pain [4, 5]. Mast cell accumulation and hyper-innervation are the two most consistent features of vestibular biopsies from patients diagnosed with vulvodynia [6, 7]. Women with a history of seasonal allergies are twice as likely to develop vulvodynia compared to allergy-free age-matched controls [8]. We previously provided biological plausibility for this association, demonstrating that single and triple labiar skin exposure to hapten oxazolone in pre-sensitized ND4 Swiss mice led to transient tactile sensitivity and an increase in cutaneous nerve density [9]. As versatile immune regulators, mast cells contribute to a broad range of acute and chronic pain responses [10] and functionally associate with nerves in a variety of patho-physiologies [11]. Whether mast cell-mediated allergic responses can drive tissue changes that provoke prolonged painful sensations remains unknown. Here, we investigated the long-term effects of repeated oxazalone exposure on the labia of ND4 Swiss mice, local and systemic inflammatory changes, and the role of mast cells in the persistence of sensitivity to touch and localized hyper-innervation.

## Methods

### Animals

#### Ethics statement

This study was carried out in accordance with the Guide for the Care and Use of Laboratory Animals of the National Institutes of Health. The protocols were approved by the Macalester Institutional Animal Care and Use Committee (IACUC protocols B13S1 and B16S2). Mice were euthanized via 100% CO_2_ inhalation at predetermined time points, and all efforts were made to minimize suffering.

6–12 week old female ND4 Swiss mice (Harlan Laboratories, Indianapolis, IN) were housed with a 12-hour light/dark cycle and free access to food and water.

#### Oxazolone treatment

**Fig 1:**
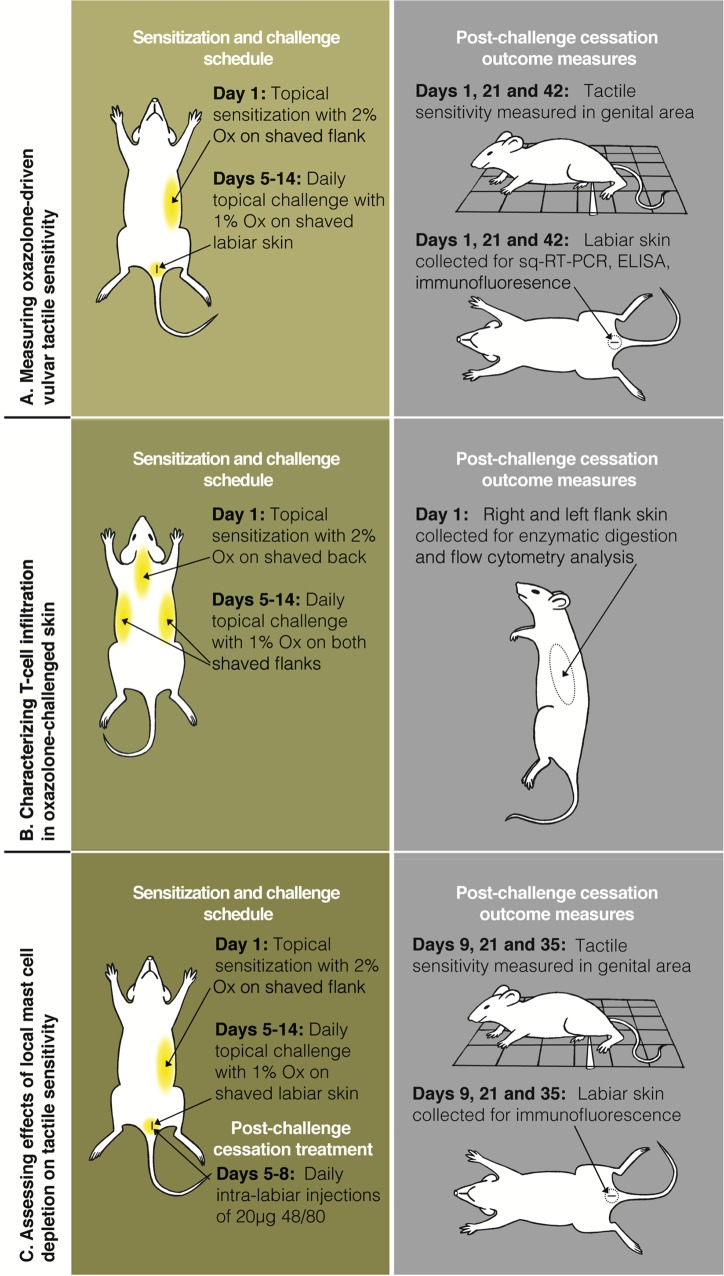
**Timeline of oxazolone sensitization, challenge, and post-challenge outcome measures** (A) To measure oxazolone-driven vulvar tactile sensitivity, mice were topically sensitized with 2% Ox on the shaved flank (day 1) and subsequently challenged on the shaved labiar skin (days 5–14) with 1% Ox or EtOH vehicle for a total of 10 challenges. Tactile sensitivity was assessed in the ano-genital ridge area 1, 21, and 42 days after challenge cessation. Labiar skin was harvested at these time points from a separate cohort of mice for assessing molecular and cellular changes in the tissue. (B) To characterize T cell infiltration in Ox-challenged skin, mice were topically sensitized on their shaved back with 2% Ox (day 1) and challenged on both shaved flanks with 1% Ox or EtOH (days 5–14). Flank skin was harvested from both sides 1 day after challenge cessation for flow cytometric analysis of T cell infiltration. (C) To assess the effects of local mast cell depletion on Ox-induced tactile sensitivity and hyperinnervation, mice were topically sensitized with 2% Ox on the shaved flank (day 1), challenged on the shaved labia with 1% Ox or EtOH (days 5–14) and treated with intralabiar injection of saline or c48/80 (days 5–8 after Ox challenge cessation). Tactile sensitivity, mast cell levels and innervation were assessed 9, 21, and 35 days after the final Ox challenge.

Mice were sensitized with topical application of 100 μL of 2% oxazolone (Ox; 4-Ethoxymethylene-2-phenyl-2-oxazolin-5-one, Sigma-Aldrich, St. Louis, MO) on the shaved flank (applied to 15 mm x 15 mm of skin) and subsequently challenged topically on the shaved genital skin around and including the labia (5 mm x 5 mm) with 40 μL of 1% Ox or ethanol vehicle daily for 10 days beginning on day 5 after sensitization (Fig 1A and 1C) (adapted from [9, 12]). Areas of topical Ox application on flank and genital skin were shaved 5 days prior to sensitization.

In separate experiments where sensitivity was not assessed, mice were sensitized with topical application of 100 μL of 2% oxazolone on a 15 mm x 15 mm area of the shaved back and subsequently challenged daily for 10 days on 25 mm x 25 mm areas of both shaved flanks with 100 μL of 1% Ox or ethanol vehicle. Flank challenges began 4 days after sensitization and were performed to obtain larger cell yields for tissue culture and intra-cellular cytokine/transcription factor staining for flow cytometry (Fig 1B).

### Mast cell depletion

Ox-sensitized and -challenged mice received intra-labiar injections of mast cell degranulator compound 48/80 (c48/80; 20 μg/mouse in 40 μl 0.9% saline; 20 μl/labium; Sigma-Aldrich) daily from day 5–8 after the cessation of Ox challenges to locally deplete mast cells in the labiar tissue (adapted from [13]; Fig 1C). Mast cell depletion was confirmed by immunofluorescence 1 day after the last c48/80 injection.

### Tactile sensitivity

We measured tactile sensitivity in a 2 mm x 2 mm area of the ano-genital ridge of mice, the posterior, hairless portion of the vulva located dorsally from the introitus, as previously described in models of vaginal candidiasis [14] and transient labiar contact hypersensitivity [9]. We measured baseline tactile sensitivity of the ano-genital ridge 48 and 24h before Ox sensitization using an electronic Von Frey anesthesiometer (IITC Life Sciences, Woodland Hills, CA) as previously described [9]. Mice were habituated to the testing conditions for 15 minutes before sensitivity measurements, as previously described [15]. In ND4 mice used in these experiments, baseline thresholds ranged from 0.5 to 1.68 g. Baseline and experimental withdrawal thresholds at 1, 21 and 42 days after Ox- and ethanol challenges and at serial time points after mast cell depletion are shown in S1 Table. As previously described [9] mice with thresholds lower than 0.50 g and mice with the two baseline measurements differing by >1.00 g were excluded. Mice were assigned to treatment groups such that the average group baseline values were similar.

Sensitivity was assessed at the same site (ano-genital ridge) and by the same investigator on days 1, 21 and 42 following challenge cessation. Average post-challenge withdrawal threshold values (in grams) were subtracted from average baseline values for each mouse. Percent decrease from baseline was then calculated for each treatment group as (change in withdrawal threshold/baseline withdrawal threshold*100). After mast cell depleting compound 48/80 was injected, sensitivity was assessed on days 9, 21 and 35 after the cessation of Ox challenges.

### Immunofluorescent staining and microscopy

Flash frozen labiar skin samples collected from CO_2_-euthanized mice on days 1, 9, 21, and 42 after challenges were embedded in Optimal Cutting Temperature compound (Sakura Finetek, Torrance, CA) and cut to obtain 10μm sections. These were fixed and stained with a primary rabbit polyclonal antibody against calcitonin gene related peptide (CGRP; Abbiotec, San Diego, CA; 1:500) and AlexaFluor 488-conjugated secondary antibody (Thermo Fisher Scientific, Wilmington, DE; 1:1000) as previously described [9, 16]. To stain mast cells, slides were incubated for one hour with FITC-Avidin (Vector Laboratories, Burlingame, CA) as previously described [9, 16]. Stained slides were cover-slipped with the anti-fade mounting medium Vectashield containing 1.5 μg/mL 4',6-diamidino-2-phenylindole (DAPI; Vector Laboratories); the DAPI stained the nuclei in tissue sections. Composite images of 10 optical 1μm sections projected on the z-axis were taken using a laser scanning confocal microscope (Olympus FV1000) and analyzed using FluoView FV1000 image analysis software (Olympus Corporation, Center Valley, PA). Mast cell and CGRP^+^ nerve density values were determined by fluorescent pixel intensity measurements taken in four representative 5000μm^2^ regions of interest in each of the three sections per slide for three slides per mouse and 4–6 mice per treatment group.

### Flow cytometry

For these experiments, mice were sensitized on the back with Ox and challenged on the flank as described above. Leukocytes were isolated from flank skin samples from CO_2_-euthanized mice as previously described [12] 1 day after the cessation of challenges (Fig 1B). Cells were washed, blocked with supernatant from a 2.4G2 hybridoma cell line (50μl, HB197TM, American Type Culture Collection, Manassas, VA), stained with fluorochrome-conjugated monoclonal antibodies (S2 Table) at 1:100 dilutions for 30 minutes and analyzed on a LSR Fortessa X-20 (Becton Dickinson, Franklin Lakes, NJ) flow cytometer. Acquired data were analyzed using FlowJo software (Version X, FlowJo LLC, Ashland, OR). Dead cells were excluded using Ghost Dye-BV510 (Tonbo Biosciences, San Diego, CA). Flank skin cells were stained with antibodies against surface proteins, fixed and permeabilized (eBioscience, San Diego, CA; manufacturer’s directions) before staining with anti-FoxP3-FITC (S2 Table). Flank skin cells were cultured in RPMI (Thermo Fisher Scientific) supplemented with 10% fetal calf serum (Serum Source International, Charlotte, NC), glutamine and antibiotics (Thermo Fisher Scientific) with PMA/ionomycin and Brefeldin A (Tonbo Biosciences) for 18 hours. Cells were then washed, surface stained as described above, fixed and permeabilized (Cytofix/Cytoperm kit, BD Biosciences, San Jose, CA) before intracellular staining with anti-IFN-γ-PE (S2 Table).

### RNA isolation and quantification of gene expression

Total RNA was extracted from flash frozen labiar skin samples collected at indicated time points using the Total RNA Mini Kit (Midwest Scientific, St. Louis, MO), quantified on a NanoDrop ND-1000 Spectrophotometer (Thermo Fisher Scientific) and reverse-transcribed using the Superscript III First-Strand Synthesis System (Thermo Fisher Scientific) in a 2720 Thermal Cycler (Thermo Fisher Scientific). Relative abundances of transcripts of interest were quantified by running 40 cycles of semi-quantitative polymerase chain reaction on a StepOnePlus thermocycler (Thermo Fisher Scientific) using quality controlled TaqMan Gene Expression Assay Primer/Probe Sets (S3 Table) and Master Mix (Thermo Fisher Scientific) and normalized to expression of housekeeping gene β-2 microglobulin to determine fold expression values for genes of interest [17].

### Protein and histamine quantification

Flash-frozen skin samples collected at indicated time points were homogenized in cell lysis buffer (Cell Signaling Technology, Beverly, MA) with protease inhibitor (EMD Millipore, Billerica, MA) using a Tissue-Tearor (BioSpec Products, Bartlesville, OK), incubated on ice for 20 minutes, strained through a 40 μm filter, and centrifuged for 10 minutes at 2000 rpm at 4°C. IFN-γ content in labiar tissue lysates was measured using an IFN-γ DuoSet ELISA kit (R&D Systems, Minneapolis, MN) and normalized to total protein content determined using the detergent-compatible DC Protein Assay (Bio-Rad, Hercules, CA). Total serum Immunoglobulin E (IgE) content was measured by ELISA (BD Biosciences) in serum isolated from blood collected either from the sub-mandibular vein or by post-mortem heart puncture, or in vaginal lavage fluid collected by gently flushing the vaginal canal with 60μl of phosphate buffered saline. We quantified histamine content of skin lysates by ELISA (Neogen Corporation, Lansing, MI). Absorbances were recorded with a PowerWave XZ microplate spectrophotometer (BioTek Instruments, Winooski, VT).

### Statistical analysis

Data were processed using Excel (Microsoft, Redmond, WA) and graphed using PRISM 5.0 (GraphPad, San Diego, CA). One-way ANOVA, *post hoc* Tukey HSD analyses, or unpaired Student’s t-test were run using JMP Software to compare treatment groups at designated time points (v. 10, SAS, Cary, NC). Two-way ANOVA with interaction and repeated measures was also performed to determine the effect of time, treatment, and interaction of time and treatment using JMP software.

## Results

### Ten oxazolone challenges to the labiar skin of sensitized ND4 swiss mice provoke a heightened and persistent tactile sensitivity

**Fig 2:**
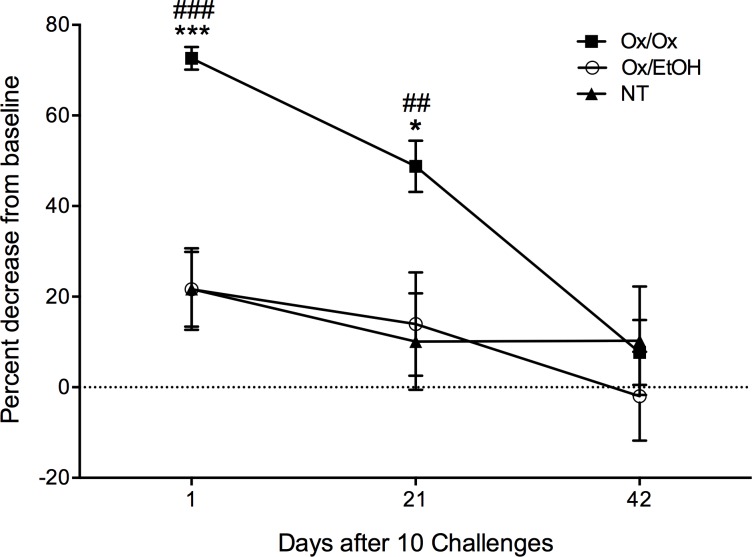
Ten oxazolone challenges provoke tactile sensitivity that persists for 21 days after cessation of challenges. Sensitized mice that received ten daily Ox challenges on the labiar skin had increased tactile sensitivity in their ano-genital ridge area compared to controls. Percent decrease in labiar withdrawal threshold for each treatment group is displayed as mean ± SEM. Significance at each time point was determined using one-way ANOVA and Tukey Kramer *post hoc* analysis based on comparisons to previously sensitized mice challenged with EtOH (Ox/EtOH; * = p<0.05, *** = p<0.001) and untreated controls (NT; ## = p<0.01, ### = p<0.001). n = 9–12 mice per treatment group; data represent two independent experiments. Raw withdrawal thresholds at baseline and post Ox-challenge cessation for each animal are shown in S1 Fig and summarized in S1 Table.

One day after cessation of ten daily allergen challenges, Ox-sensitized female ND4 Swiss mice had a 70% decrease in withdrawal threshold to light pressure applied with an electronic Von Frey meter when compared to their baseline sensitivity (Fig 2A). Both shaved, untreated mice and sensitized mice challenged with ethanol (EtOH; vehicle) were significantly less sensitive to touch than Ox-treated mice, with only a 20% decrease in withdrawal thresholds compared to their baselines. Individual withdrawal thresholds for each mouse in all treatment groups are shown in supporting data (S1A–S1C Fig). Ox-challenged mice were significantly more sensitive to pressure compared to control groups at 1 and 21 days after the final challenge; all groups returned to baseline sensitivity by 42 days after cessation of allergen challenges (Fig 2A); the same mice were evaluated at all time points. When we fit the data to a two-way repeated measures ANOVA model, we found that both Ox-treatment (p<0.0001) and time (p<0.0001) had a significant effect on sensitivity. No significant random effects were found. We also investigated an interaction of time and treatment. The interaction term approached, but did not reach, statistical significance (p = 0.0538). This pronounced, persistent sensitivity was localized to the site of allergen challenge; tactile sensitivity at a distal site (the hind paw) remained unchanged after labiar Ox challenges (S1D Fig, S4 Table). Previously, we showed that baseline tactile sensitivity is not modulated by stages of the estrus cycle [9]. Here, we also show that Ox-triggered tactile sensitivity is not affected by estrus cycle stage (S1E Fig, S4 Table). Furthermore, we detected no overt signs of inflammation or tissue injury by visual inspection 21 days after 10 Ox challenges, when sensitivity was still detectable and significant over vehicle-challenged controls (S1F Fig).

### Ten oxazolone challenges to the labiar skin of sensitized ND4 swiss mice result in sustained sensory hyper-innervation at the site of allergen exposure.

**Fig 3:**
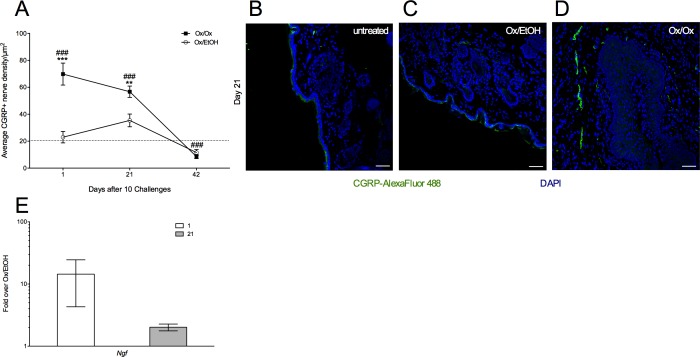
Labiar CGRP^+^ nerve density is increased after oxazolone challenges accompanying an increase in *Ngf* transcripts. (A) Density of CGRP^+^ nerve fibers in 10 μm labiar skin cryo-sections from sensitized mice challenged with Ox or EtOH, displayed as mean ± SEM (n = 3-5/treatment group). Dashed line corresponds to average CGRP^+^ nerve fiber density/μm^2^ in untreated mice. Images are representative from day 21 after cessation of challenges (B-D; 20x magnification; scale bar represents 50 μm). Means compared to Ox/EtOH (** = p<0.01, *** = p<0.001) or untreated controls (### = p<0.001) at each time point; significance determined by one-way ANOVA and Tukey Kramer *post hoc* analysis. (E) Relative abundance of *Ngf* in Ox- vs. EtOH-challenged mice 1 day after 10 challenges displayed as mean ± SEM (n = 5-6/treatment group; two independent experiments).

ND4 female mice showed an increase in CGRP^+^ sensory neurons at the site of Ox challenge along with heightened sensitivity to touch. Ox-challenged mice presented a significant, >3-fold increase in cutaneous labiar CGRP intensity compared to vehicle-challenged and untreated controls 1 day after 10 Ox challenges (Fig 3A). Significant hyper-innervation persisted in Ox-challenged mice at least until day 21 (Fig 3A–3D); all groups returned to baseline levels by day 42. Thus, significant hyper-innervation was present for three or more weeks after allergen challenges had ceased, but it was no longer evident by six weeks after the end of Ox treatment. Transcripts encoding nerve growth factor (NGF) were elevated ~10-fold in Ox-challenged mice over EtOH-treated controls in the labiar skin tissue 1 day after 10 Ox challenges, suggesting that the local tissue environment in the allergic site supported increased growth and maintenance of neurons; this increase was less pronounced by day 21 (Fig 3E).

### Ten oxazolone challenges to the labiar skin of sensitized ND4 swiss mice induce local accumulation of mast cells.

**Fig 4:**
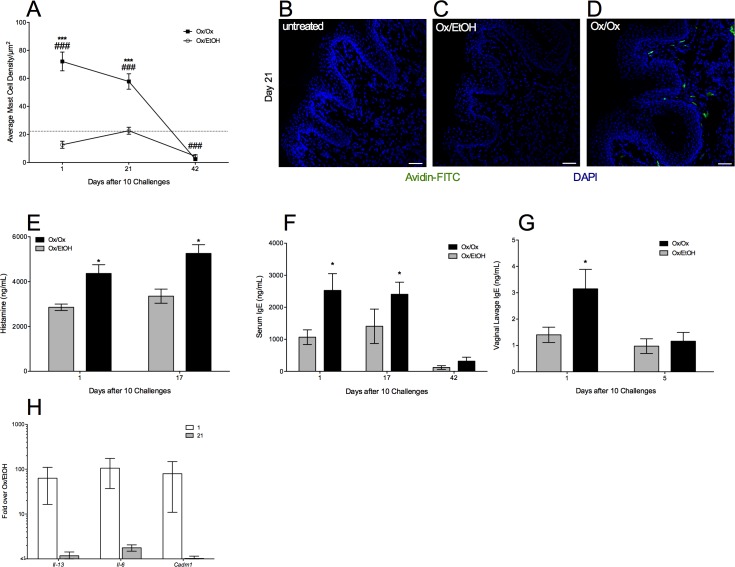
Labiar mast cell density is increased after oxazolone challenges accompanying an increase in modulatory factors. (A) Density of Avidin^+^ mast cells in 10 μm labiar skin cryo-sections from sensitized mice challenged with Ox or EtOH, displayed as mean ± SEM (n = 4-6/treatment group). Dashed line corresponds to average mast cell numbers/μm^2^ in untreated animals. Images are representative from day 21 after challenge cessation (B-D; 20x magnification; scale bar represents 50 μm). Means are compared to Ox/EtOH (*** = p<0.001) or untreated controls (### = p<0.001) at each time point; significance determined using one-way ANOVA and Tukey Kramer *post hoc* analysis. (E) Tissue histamine content in labiar skin of Ox- and EtOH-challenged mice 1 and 21 days after cessation of oxazolone challenges. Total IgE content in serum (F) and vaginal lavage (G) in Ox vs. EtOH-challenged mice at indicated time points after sensitization. (H) Relative abundance of *Il13*, *Il6*, and *Cadm1* in Ox- vs. EtOH challenged mice 1 day after 10 oxazolone challenges displayed as mean ± SEM (n = 5-6/treatment group; two independent experiments).

Mast cell numbers in Ox-challenged skin were 4-fold higher than in ethanol-challenged skin on 1 day after the cessation of Ox challenges (Fig 4A) and remained elevated until day 21 (Fig 4A–4D); all groups resolved to baseline by day 42. Total histamine levels in the labiar skin of Ox-treated mice were significantly higher than in ethanol-treated mice (Fig 4E) possibly reflecting the increased numbers of histamine containing mast cells at the allergic site. Similarly to the observed pattern of hyper-innervation, increased mast cell accumulation at the site of Ox challenge was clearly present at least for three weeks after allergen challenges were completed, but resolved to baseline levels by six weeks post challenge cessation.

Elevated circulating IgE is a hallmark of atopic disease [18]. Serum IgE levels were significantly increased in Ox- over ethanol-challenged mice (Fig 4F) on day 1 after 10 Ox challenges and remained elevated over two weeks. Total IgE in the vaginal lavage increased slightly but significantly in the Ox-challenged mice 1 day after 10 Ox challenges (Fig 4G). IgE can enhance mast cell survival, acting in part through IL-6 and IL-13 signaling [19]. We therefore tested the levels of *Il6* and *Il13* mRNAs. Both transcripts were elevated in labiar skin ~100-fold 1 day after 10 Ox challenges (Fig 4H). This increase was no longer discernible by day 21 for *Il13* although *Il6* transcripts remained slightly elevated in Ox-challenged mice.

The cell adhesion molecule 1 (CADM1) regulates mast cell-neuron synapses in atopic dermatitis [20]. *Cadm1* transcripts were elevated ~100 fold in Ox-challenged over vehicle-challenged labiar skin 1 day after 10 Ox challenges supporting the concomitant increase of nerve density and mast cells at the allergic site; this increase was no longer discernible on day 21 (Fig 4H).

### Local mast cell depletion after repeated exposure to allergen reduces innervation and tactile sensitivity.

**Fig 5:**
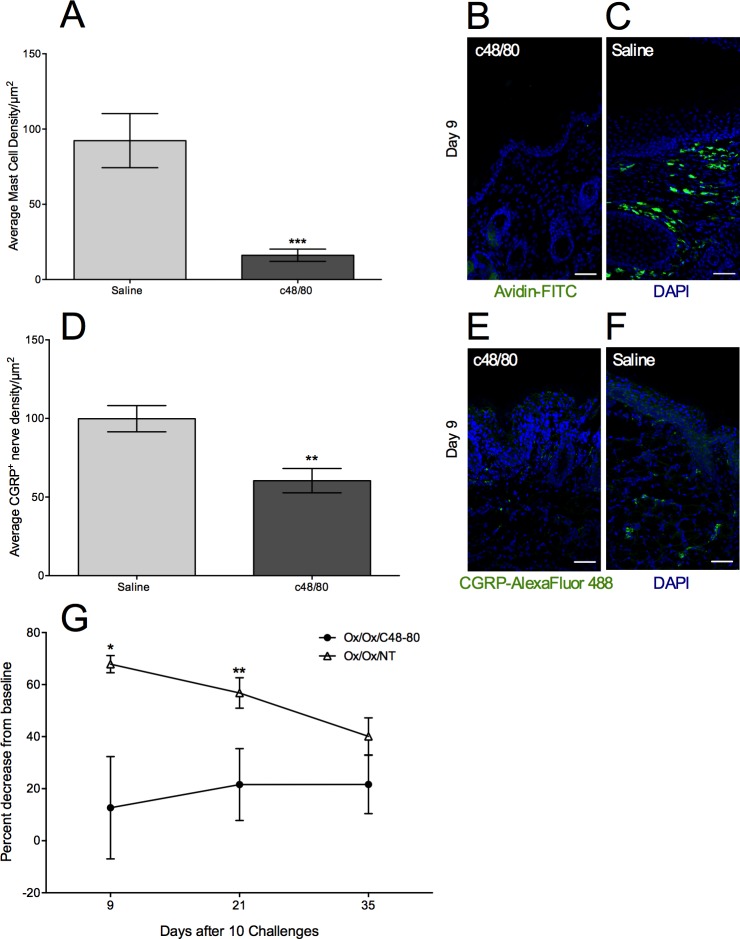
Injection of c48/80 after challenges depletes mast cells and reduces CGRP^+^ nerve density and sensitivity. Density of Avidin^+^ mast cells (A) and CGRP^+^ cutaneous nerves (D) on day 9 after 4 treatments with c48/80 or saline (administered on days 5–8 after cessation of 10 Ox challenges) in 10 μm labiar cryo-sections, displayed as mean ± SEM (n = 2-3/treatment group). Representative images for mast cells (B-C) and nerves (E-F); 20x magnification; scale bar represents 50 μm. Means are compared to Ox/EtOH (** = p<0.01, *** = p<0.001); significance determined using one-way ANOVA and Tukey Kramer *post hoc* analysis. (G) Tactile sensitivity in mice treated with either saline or c48/80 (n = 6–9 mice per treatment group; two independent experiments). Means are compared to Ox/Ox/Saline (* = p<0.05, ** = p<0.01, *** = p<0.001); significance determined using an unpaired Student’s T test at each time point.

ND4 mice were treated with daily injections of mast cell degranulator c48/80 (10 μg/labium) on days 5–8 after Ox challenge cessation (Fig 1C). To confirm depletion, we assessed mast cell density in the labiar skin on day 9 after 10 Ox challenges i.e. 1 day after c48/80 injections were completed, and detected >5-fold decrease (Fig 5A–5C). Mast cell depletion was accompanied by a significant ~2-fold reduction in the density of cutaneous CGRP^+^ neurons in the allergic skin (Fig 5D–5F) and a significant reduction in tactile sensitivity assessed at individual time points on days 9 and 21 (Fig 5G) compared to Ox-challenged mice that were not treated with c48/80. When we fit the data to a two-way repeated measures ANOVA model, we found that c48/80 injections had significant effect on sensitivity (p = 0.0069). The effect of time was not statistically significant, nor did we find evidence of a random effect. We investigated an interaction of time and c48/80 treatment, but the interaction term did not reach statistical significance. Mice given intra-labiar injections of saline (vehicle) had indistinguishable sensitivity from Ox-sensitized and challenged controls 9 days after the cessation of Ox challenges (S2 Fig).

### Regulatory CD4 T cells and IFN-γ producing CD8 resident memory cells accumulate in the affected skin after multiple Ox challenges.

**Fig 6:**
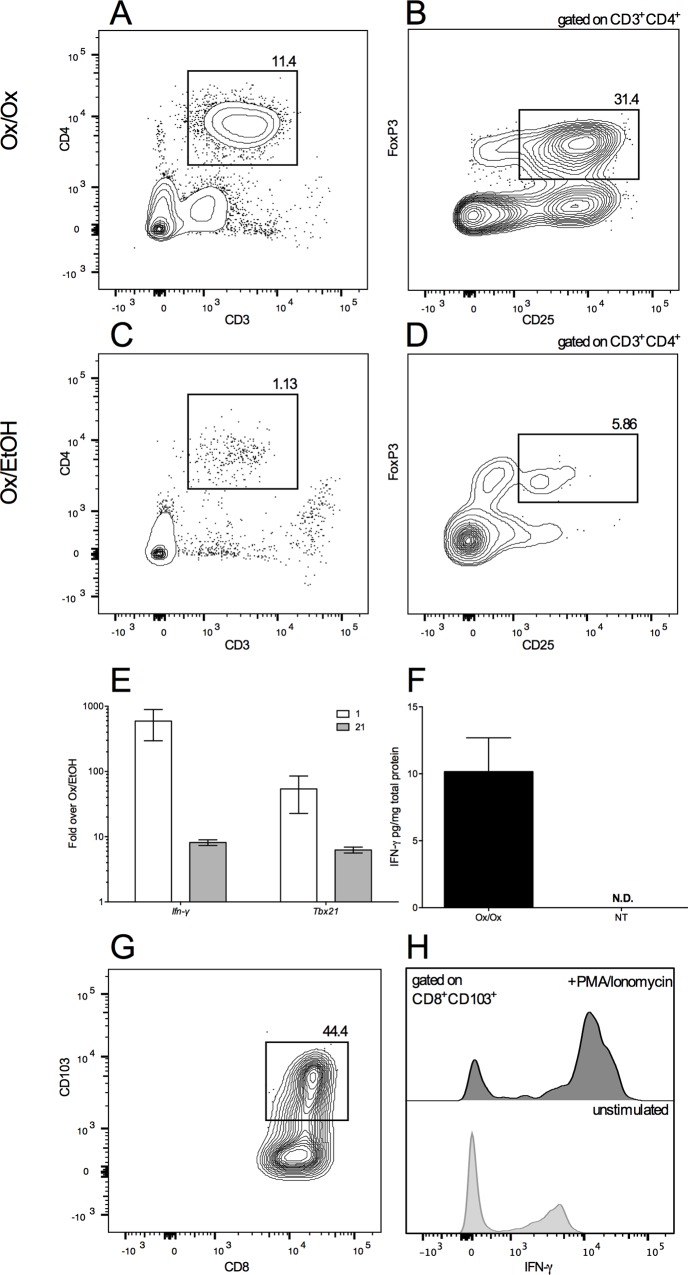
CD4^+^CD25^+^FoxP3^+^ T cells and IFN-γ producing CD8^+^CD103^+^ memory T cells accumulate in oxazolone challenged skin. Flow cytometric analysis of collagenase-digested, gradient-separated flank skin cells from Ox- (A-B) or EtOH- (C-D) challenged mice 1 day after the cessation of 10 challenges. Cells in (A) and (C) are scatter-gated for lymphocytes, single cells, live, and CD45^+^. Data are pooled from 10 mice per treatment group. Relative abundance of *Ifn-γ* and *Tbx21* (E) and total IFN-*γ* protein content (F) in labiar skin of Ox- vs. EtOH challenged mice after 10 challenges displayed as mean ± SEM (n = 5-6/treatment group). (G-H) Flow cytometric analysis of collagenase-digested, gradient-separated flank skin cells collected 1 day after 10 challenges from Ox-challenged mice and cultured for 18 hours with or without PMA/ionomycin; cells in (G) are live, singlet-gated, scatter-gated for lymphocytes, and CD45^+^CD3^+^CD8^+^.

Regulatory T cells (T_reg_) recruit mast cell progenitors to mouse airways after intra-nasal ovalbumin challenge [21] and promote focal mastocytosis in a mouse model of hereditary colon cancer [22]. With this in mind, we examined T_reg_ accumulation in Ox-challenged skin. As Ox-challenged labiar skin yields very few cells after enzymatic digestion, we set up experiments where mice were sensitized on the back with 2% Ox and challenged on both flanks with 1% Ox (Fig 1B) to obtain larger Ox-challenged skin samples. One day after 10 Ox challenges, flow cytometric analysis of flank skin from Ox-sensitized and challenged ND4 mice showed an accumulation of CD3^+^CD4^+^ cells, ~32% of which were CD25^+^FoxP3^+^. Few CD4^+^CD25^+^FoxP3^+^ cells were detected in vehicle-challenged mice (Fig 6A–6D). Approximately 625 CD4^+^CD25^+^FoxP3^+^ cells were recovered from 25 mm x 25 mm of flank skin of each mouse challenged with Ox compared to <60 CD4^+^CD25^+^FoxP3^+^ cells detected in skin samples from vehicle-challenged mice.

IFN-γ signaling drives mast cell contributions to chronic asthma-like tissue remodeling seen in ovalbumin-challenged mice [23]. Transcripts encoding IFN- were elevated ~500-fold in Ox vs. vehicle-challenged labiar skin 1 day after 10 Ox challenges and remained elevated ~10-fold over controls at day 21 (Fig 6E). *Tbx21* mRNA encoding T-bet, a known driver of IFN-γ production in multiple cell types [24], was also increased ~100-fold in Ox-challenged over vehicle-challenged labiar skin on day 1, and remained elevated ~10-fold at day 21 (Fig 6E). On day 1, total IFN-γ protein levels were also significantly increased in Ox-challenged vs. untreated labiar skin (Fig 6F). CD3^+^CD8^+^CD103^+^CD44^+^ resident memory cells [25] isolated from the flank skin after 10 Ox challenges and cultured for 18 hours with and without PMA/ionomycin stimulation produced detectable IFN-γ, suggesting they could be potential sources of IFN-γ *in vivo* (Fig 6G and 6H).

## Discussion

Ten labiar Ox challenges in sensitized ND4 mice provoked increased tactile sensitivity that lasted over three weeks after challenge cessation, and resulted in a marked accumulation of mast cells and overgrowth of CGRP^+^ sensory neurons at the site of allergen challenge. Painful sensitivity to touch, mast cell increases, and nerve overgrowth in the absence of overt inflammation are consistent with the most common characteristics of localized, provoked vulvodynia [2]. A diagnosis of vulvodynia is made in the clinic by reporting painful sensations in response to palpation of the vestibule, mons, labia, perianal and perineal areas by applying light consistent pressure with a cotton swab, similar to our method of measuring responses to pressure in the ano-genital ridge of mice [5]. While some vulvodynia patients report referred peripheral sensitivity in non-vestibular sites [26], we did not find altered sensitivity in the hind paw tissue of mice with lowered labiar withdrawal threshold for pressure. Kakurai et al. reported mast cell-dependent nerve elongation after a single Ox challenge in sensitized C57BL/6 mice [16]. Earlier, we showed that nerve density increases within 48 hours after one or three Ox challenges [9]. Here, we found that after 10 Ox challenges, local nerve density increased 3–4 fold and remained elevated for over three weeks. This allergen-provoked local hyper-innervation in mice sensitive to touch and pressure parallels the increase in vestibular innervation seen at the painful sites of women diagnosed with vulvodynia [7].

Mast cells accumulate in affected tissues of C57BL/6 mice following repeated Ox challenges [16, 27, 28]. We noted a concomitant ~4-fold increase in the abundance of tissue mast cells at day 1 and a ~2-fold increase in total histamine content that persisted through day 21 after 10 labiar Ox challenges. Increases in mast cell numbers and degranulation [6, 29] as well as secondary mast cell hyperplasia [30] have been reported in vulvar biopsies of women diagnosed with vulvodynia. We are currently investigating the extent of mast cell activation and degranulation at the sensitive sites.

Mast cell-nerve interactions contribute to multiple inflammatory pathologies [10, 11]. Mast cells can produce NGF in both rodents [31] and humans [32]. Increased CADM1 on mast cells has been identified in atopic dermatitis and pulmonary emphysema [33] and has been shown to enhance nerve-mast cell interaction in a mouse model of trinitrochlorobenzene-driven contact hypersensitivity [20]. Here, we found significantly elevated *Ngf* and *Cadm1* mRNAs in touch-sensitive, allergen-challenged mice over vehicle-treated controls on 1 day after 10 Ox challenges. However, these differences were less pronounced by day 21, suggesting that these are early markers of tissue changes that support the increased innervation facilitating prolonged sensitivity. Local changes in NGF and CADM1 have not been reported in vulvodynia patients. However, patient samples are usually acquired after symptoms have been established for some time, and these molecular changes may not be detectable later. Levels of total IgE antibodies were significantly increased in both the serum and vaginal lavage of Ox-challenged mice. In one study, ~30% of vulvodynia patients presented elevated vaginal IgE including 16 women with IgE antibodies against seminal fluid antigens [34]. Differences in serum IgE have not been assessed in vulvodynia patients to date. Circulating IgEs are a survival factor for mast cells acting via IL-6 and IL-13 [16]. We detected increased levels of transcripts encoding both of these cytokines in the labiar skin of Ox-challenged but not vehicle-challenged mice.

The characteristics of inflammatory pathways that contribute to vulvodynia pathology remain poorly understood [35]. TNF-α and IL-1β are elevated in vestibular tissue homogenates from vulvodynia patients [36]. Additionally, vulvar fibroblasts derived from patients as opposed to controls produce larger amounts of IL-6 and IL-8 when challenged with yeast antigen [37], and are responsive *in vitro* to low doses of *Candida albicans* in a Dectin-1-dependent manner [38]. Yeast infections can predispose to vulvodynia [39] and others have shown increased ano-genital sensitivity in mice following repeated vulvovaginal candidiasis [14]. In our study, transcripts encoding TNF-α and CXCL-2 (the murine homolog of human IL-8) were also increased relative to controls in Ox-challenged mice (S3 Fig). CXCL2 protein levels were elevated in experimental vs. untreated mice (S3 Fig). Given the high expression of *Cxcl2* mRNAs and protein in Ox-treated mice, we measured neutrophil influx into the affected skin since CXCL-2 is a potent attractant for these cells [40] but found no changes in levels of activated neutrophils (S3 Fig).

Ectopic germinal center-like structures containing T cells, B cells, macrophages and dendritic cells have been reported in vestibular tissue from vulvodynia patients [41]. We detected T cell accumulation in Ox-challenged murine skin suggesting an overall inflammatory profile. Regulatory T cells have been shown to be required for mast cell progenitor recruitment into allergic airways of ovalbumin-challenged mice [21]. Ox-challenged but not vehicle-treated mice in our experiments show distinct infiltration of CD4^+^CD25^+^FoxP3^+^ cells that could, in turn, recruit mast cells into affected labiar skin. Mast cells also require IFN-γ signaling to mediate immune infiltration and pulmonary remodeling in a mouse model of chronic asthma [23]. IFN-γ transcripts showed the most substantial and sustained increase in Ox-challenged labiar skin over controls in our experiments. One day after the last allergen challenge, IFN-γ protein levels were also increased ~10-fold over untreated controls. *Tbx21* mRNAs encoding T-bet–a potent driver of IFN-γ production–were also increased in labiar tissue at this time. CD3^+^CD8^+^CD103^+^CD44^+^ resident memory T cells identified in Ox-challenged mice were positive for IFN-γ by intracellular staining both without stimulation and after overnight incubation with PMA/ionomycin, suggesting they could produce IFN-γ *in situ*. These findings are in agreement with a recent report showing IFN-γ^+^ resident memory CD8^+^ T cells in the ear pinnae of mice challenged monthly with Ox (for a total of three challenges) after sensitization [42]. While it remains to be seen whether mast cell-driven tissue changes in the skin are similar to those described in the airway [20], it appears that IFN-γ is present in the affected tissue, at least at an early stage. Overall, the inflammatory skin microenvironment after repeated Ox exposures has cellular and molecular characteristics that can support the recruitment and activity of mast cells that are potentially critical to the subsequent increases in innervation and tactile sensitivity. Gimenez-Rivera and colleagues showed that mast cell-deficient mice have exacerbated chronic, but not acute, contact hypersensitivity to Ox [28]. It is possible that MC accumulation at allergic sites drives the resolution of dermal inflammation after prolonged allergen exposure while potentially promoting a persistent increase in sensitivity. Farmer *et al*. reported that vaginal candidiasis-induced ano-genital sensitivity in mice was accompanied by hyper-innervation without detectable changes in immune cell status [14]. While we assay labiar skin of ectodermal origin and Farmer and colleagues targeted vaginal epithelium of mesodermal origin, clinical measurements of pain and inflammation are conducted in human vestibular tissue of endodermal origin [42]. Whether embryonic origin of tissue affects tactile sensitivity is currently unknown. In ongoing studies, we are comparing the effects of multiple hapten exposures in the vaginal epithelium and the labia.

Our findings indicate that the local tissue microenvironment supports concomitant increases in nerves and mast cells after repeated allergen challenges. Therefore, it is likely that nerve-mast cell interactions are important in the formation of the localized, provoked sensitivity to touch that ensues. When we depleted local mast cells in the labiar tissue with a short-term administration of c48/80 after challenge cessation, we found a reduction in both local sensory innervation and tactile sensitivity. While therapeutic administration of the mast cell stabilizer sodium cromolyn has been ineffective in idiopathic vulvar vestibulitis [43], we show here that mast cell depletion, rather than stabilization, can potentially reduce hyperinnervation and enhanced sensitivity to touch that occurs after repeated exposures to allergen.

Our work provides strong evidence that repeated allergen-induced mechanical sensitivity is at least in part mediated by *de novo* nerve growth at the site of challenge, and warrants further investigation into mechanisms of allergy-induced nerve sprouting that contribute to pain amplification. Others have shown sprouting of cutaneous sensory neurons in the hind paws of mice under inflammatory conditions associated with thermal and mechanical pain [44] as well as Complete Freund’s Adjuvant-induced arthritis and skin inflammation [45].

We have developed a new, clinically relevant murine model of persistent allergy-provoked tactile sensitivity driven by a mast cell-centered tissue response. Early inflammatory events set the stage for long-term mast cell increases in the tissue that then support the overgrowth and stability of sensory neurons. While the current study focuses on the biological plausibility of allergy-driven tissue changes, repeated injury or infection at a particular tissue site may also stimulate similar remodeling responses. Mechanisms underlying recruitment of mast cell progenitors in tissue remodeling are being elucidated in many models of allergic disease [46]. Since increased vestibular mast cell accumulation is one of the most consistent characteristics of vulvodynia, specifically targeting tissue-resident mast cells as well as mast cell recruitment into tissues may be viable therapeutic approaches not only for allergen-provoked vulvodynia but also for other forms of the condition epidemiologically associated with a history of infections [39] or violence-related injury [47]. Such therapeutic strategies may also limit chronic pain associated with increased accumulation and activation of mast cells in a diverse array of pathologies including cystitis [48], migraines [49] and inflammatory bowel disorders [50].

## Supporting information

S1 Fig Labiar challenges do not reduce hindpaw withdrawal thresholds; estrus stage does not affect sensitivity measurements. (A-C) Withdrawal thresholds of (A) Ox-sensitized mice challenged 10x with Ox on the labia, (B) Ox-sensitized mice challenged 10x with ethanol on the labia, and (C) untreated mice. Each colored symbol represents a longitudinally assessed animal. (D) Sensitized mice that received ten daily Ox challenges to the vulvar region have no significant change in tactile sensitivity in the hind paw footpad compared to vehicle-treated controls; percent change in withdrawal threshold of each treatment group is displayed as mean ± SEM. n = 7–8 mice per treatment group. Data fit to a two-way ANOVA with interaction and repeated measures show no significant effect of time, treatment, interaction of the two, or random effects on hindpaw sensitivity after labiar Ox challenge. (E) Vaginal lavage smears were collected from mice for four consecutive days after the cessation of 10 oxazolone challenges to the labia and stained with 0.1% crystal violet as previously described [51]. Relative proportions of nucleated epithelial cells, cornified epithelial cells, and leukocytes were quantified to assign mice to stage of cycle. No differences in Ox-provoked sensitivity were found at day 1 or 21 after challenge cessation between mice in the estrus, pro-estrus, diestrus and metestrus stages. Raw withdrawal thresholds are summarized in S4 Table. (F) Ox-challenged mice (left) show no obvious signs of inflammation at 21 days after challenge cessation, despite enhanced sensitivity to pressure at this time point. Ethanol-challenged mice (right) are shown for comparison.
(TIFF)

S2 Fig Four intra-labiar saline injections on days 5–8 after challenge cessation do not affect tactile sensitivity. Changes in labiar withdrawal threshold of Ox-challenged mice that received intra-labiar injections of 0.9% saline are not significantly different (p = 0.3766) from those seen in mice challenged with Ox alone (n = 9/treatment group). Shown here is mean ± SEM (Student’s t-test).
(TIFF)

S3 Fig Ox challenges are associated with increasedTNF-α and CXCL-2 mRNA and protein without neutrophil influx. (A) Relative abundance of *Tnf-*α and *Cxcl-2* is increased in Ox- vs. EtOH-challenged mice 1 and 21 days after 10 challenges, displayed as mean ± SEM (n = 5-6/treatment group; two independent experiments). (B) Total CXCL-2 protein content is increased in Ox- vs. untreated mice 1 day after challenge cessation measured by ELISA (R&D Systems, manufacturer’s directions) displayed as mean ± SEM (n = 4-6/treatment group). (C) Ox-, EtOH-challenged mice and untreated controls have similar myeloperoxidase activity in the labiar skin. To measure myeloperoxidase activity, samples were frozen at -80°C in 50 mM K2HPO4 buffer (pH 6.0) with 0.05% hexadecyl trimethylammonium bromide (HTAB), thawed, homogenized in 5x volumes of HTAB buffer, sonicated 3x for 10 s, frozen and thawed 3x, re-sonicated, and centrifuged for four minutes. Absorbance was recorded at 450 nm after a 20-min incubation in 50 mM phosphate buffer (pH 6.0) with 0.025% hydrogen peroxide and 0.167 mg/mL *o*-dianisidine dihydrochloride at room temperature in the dark [52]. Myeloperoxidase levels are normalized to tissue weight and displayed as OD/g of wet tissue. Shown here is mean ± SEM (n = 3/treatment group).
(TIFF)

S1 Table Labiar withdrawal threshold values of untreated, pre-sensitized Ox challenged, ethanol-challenged, and c48/80-treated Ox-challenged mice. (DOCX)

S2 Table Lineage and activation markers used to identify skin-infiltrating cells in oxazolone-challenged labia. (DOCX)

S3 Table Accession numbers for Taqman primer-probe sets (Life Technologies, Carlsbad, CA) for semi-quantitative PCR used to measure relative transcript abundance in Ox-challenged labia. (DOCX)

S4 Table Hindpaw withdrawal thresholds to pressure in mice challenged on the labia, and labiar withdrawal thresholds at different stages of the estrus cycle after Ox challenge cessation. (DOCX)
