# Supplementary figures and images for: Repeated hapten exposure induces persistent tactile sensitivity in mice modeling localized provoked vulvodynia
Source: PLoS One. 2017 Feb 3;12(2):e0169672. doi: 10.1371/journal.pone.0169672 (PMC5291437; doi:10.1371/journal.pone.0169672)

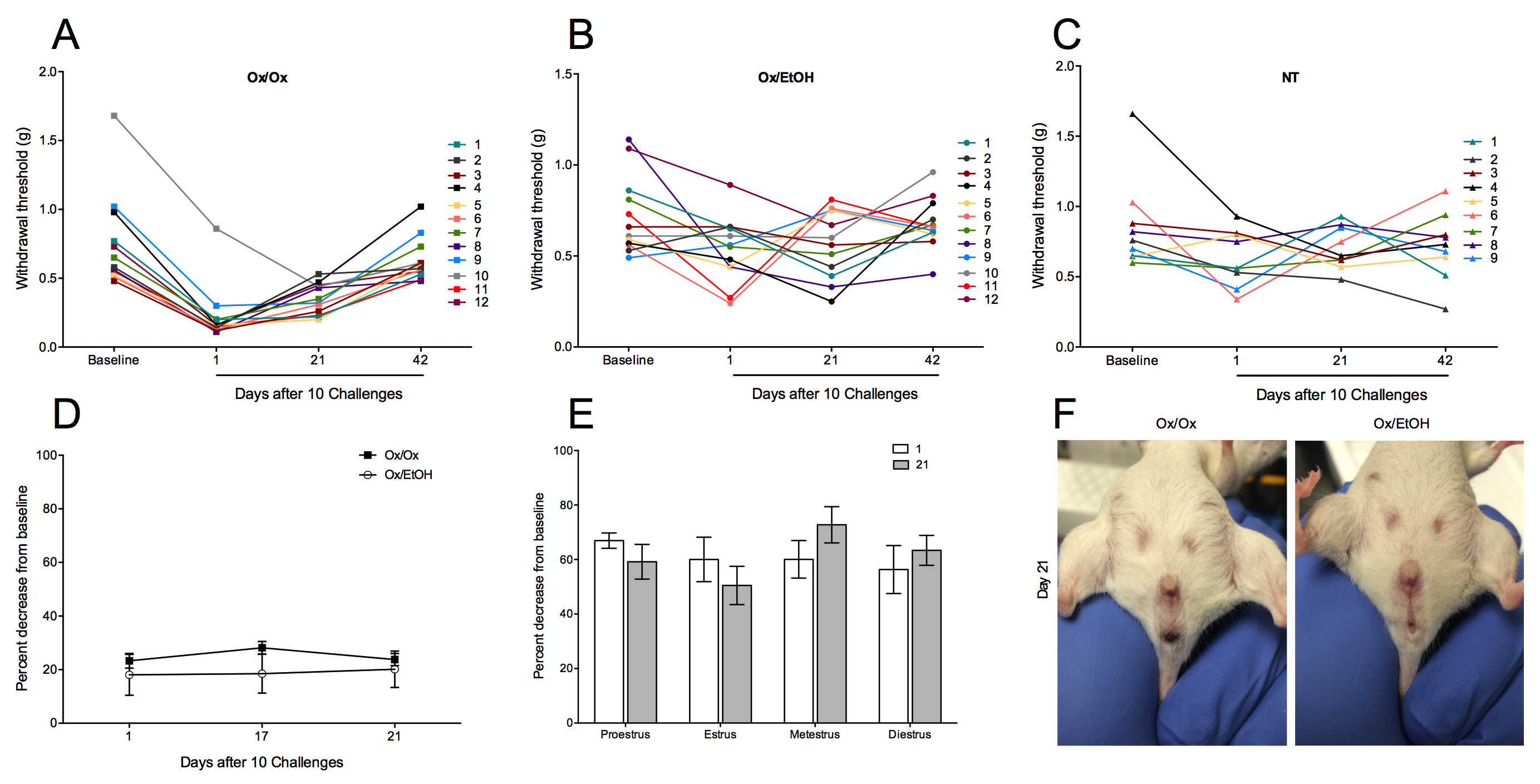

Supplement: S1 Fig — (A-C) Withdrawal thresholds of (A) Ox-sensitized mice challenged 10x with Ox on the labia, (B) Ox-sensitized mice challenged 10x with ethanol on the labia, and (C) untreated mice. Each colored symbol represents a longitudinally assessed animal. (D) Sensitized mice that received ten daily Ox challenges to the vulvar region have no significant change in tactile sensitivity in the hind paw footpad compared to vehicle-treated controls; percent change in withdrawal threshold of each treatment group is displayed as mean ± SEM. n = 7–8 mice per treatment group. Data fit to a two-way ANOVA with interaction and repeated measures show no significant effect of time, treatment, interaction of the two, or random effects on hindpaw sensitivity after labiar Ox challenge. (E) Vaginal lavage smears were collected from mice for four consecutive days after the cessation of 10 oxazolone challenges to the labia and stained with 0.1% crystal violet as previously described [51]. Relative proportions of nucleated epithelial cells, cornified epithelial cells, and leukocytes were quantified to assign mice to stage of cycle. No differences in Ox-provoked sensitivity were found at day 1 or 21 after challenge cessation between mice in the estrus, pro-estrus, diestrus and metestrus stages. Raw withdrawal thresholds are summarized in S4 Table. (F) Ox-challenged mice (left) show no obvious signs of inflammation at 21 days after challenge cessation, despite enhanced sensitivity to pressure at this time point. Ethanol-challenged mice (right) are shown for comparison. (TIFF) [file pone.0169672.s001.tiff]

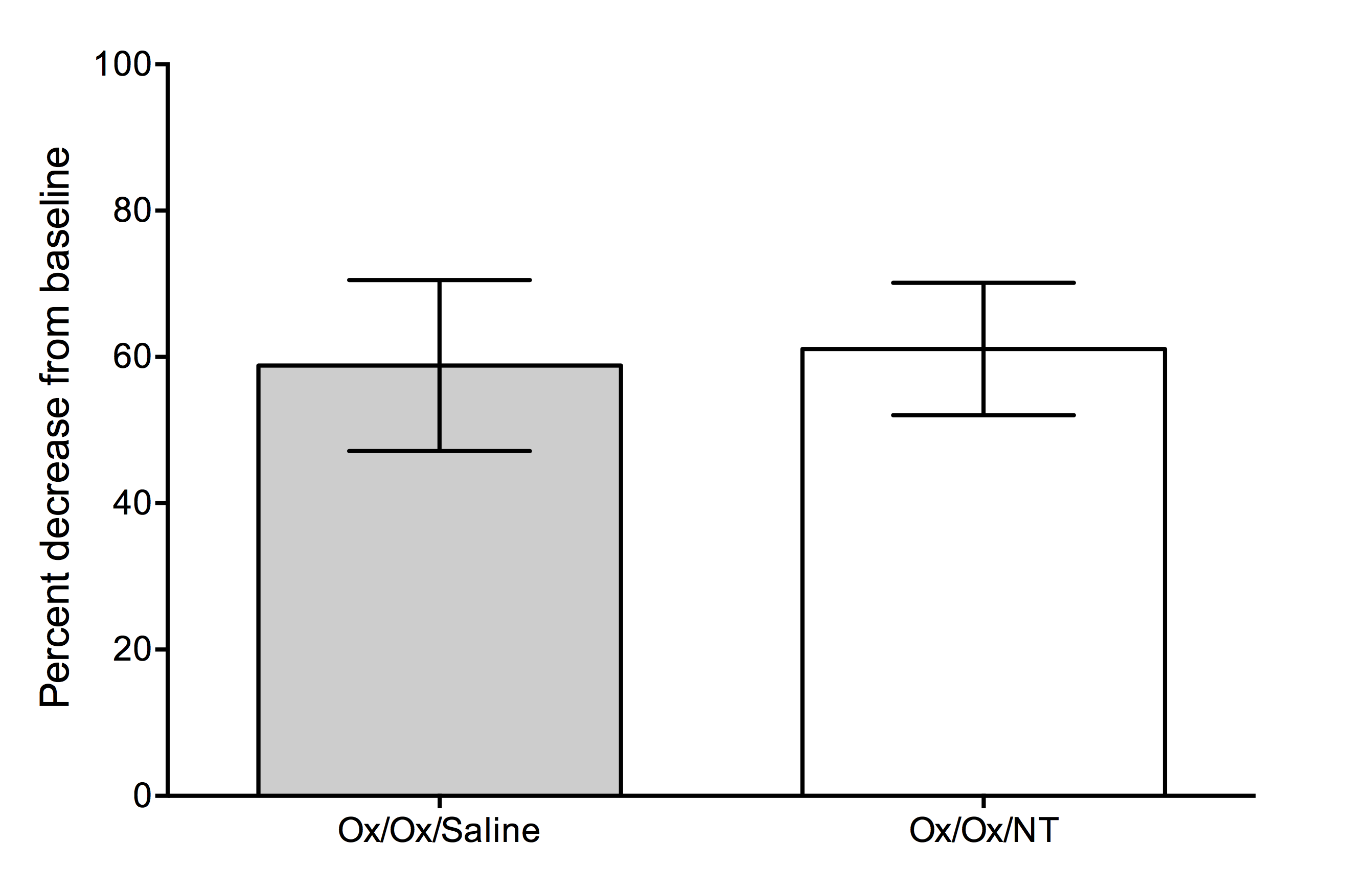

Supplement: S2 Fig — Changes in labiar withdrawal threshold of Ox-challenged mice that received intra-labiar injections of 0.9% saline are not significantly different (p = 0.3766) from those seen in mice challenged with Ox alone (n = 9/treatment group). Shown here is mean ± SEM (Student’s t-test). (TIFF) [file pone.0169672.s002.tiff]

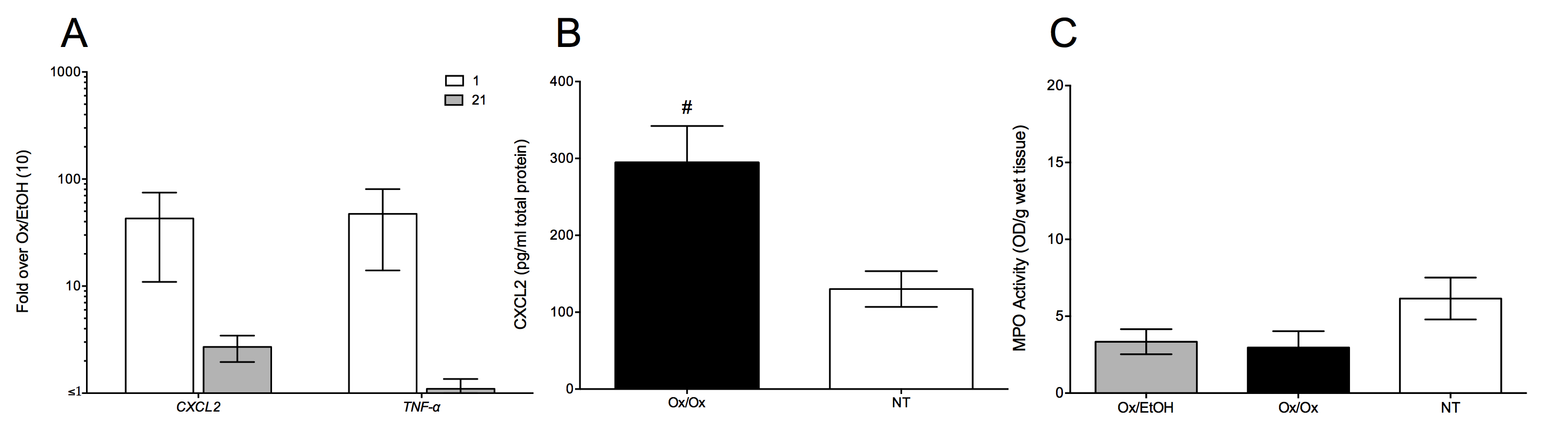

Supplement: S3 Fig — (A) Relative abundance of Tnf-α and Cxcl-2 is increased in Ox- vs. EtOH-challenged mice 1 and 21 days after 10 challenges, displayed as mean ± SEM (n = 5-6/treatment group; two independent experiments). (B) Total CXCL-2 protein content is increased in Ox- vs. untreated mice 1 day after challenge cessation measured by ELISA (R&D Systems, manufacturer’s directions) displayed as mean ± SEM (n = 4-6/treatment group). (C) Ox-, EtOH-challenged mice and untreated controls have similar myeloperoxidase activity in the labiar skin. To measure myeloperoxidase activity, samples were frozen at -80°C in 50 mM K2HPO4 buffer (pH 6.0) with 0.05% hexadecyl trimethylammonium bromide (HTAB), thawed, homogenized in 5x volumes of HTAB buffer, sonicated 3x for 10 s, frozen and thawed 3x, re-sonicated, and centrifuged for four minutes. Absorbance was recorded at 450 nm after a 20-min incubation in 50 mM phosphate buffer (pH 6.0) with 0.025% hydrogen peroxide and 0.167 mg/mL o-dianisidine dihydrochloride at room temperature in the dark [52]. Myeloperoxidase levels are normalized to tissue weight and displayed as OD/g of wet tissue. Shown here is mean ± SEM (n = 3/treatment group). (TIFF) [file pone.0169672.s003.tiff]
