# Supplementary material for: Repeated hapten exposure induces persistent tactile sensitivity in mice modeling localized provoked vulvodynia
Source: PLoS One. 2017 Feb 3;12(2):e0169672. doi: 10.1371/journal.pone.0169672 (PMC5291437; doi:10.1371/journal.pone.0169672)
Supplement: S1 Table — (DOCX) [file pone.0169672.s004.docx]

**Table S1.** Labiar withdrawal threshold values of untreated, pre-sensitized Ox challenged, ethanol-challenged, and c48/80-treated Ox-challenged mice. (Top) Shown are withdrawal thresholds (mean ± SEM; in grams) for shaved untreated (NT; n = 9), or Ox-sensitized ND4 mice challenged on the labia 10 times with either vehicle (Ox/EtOH (10); n = 12) or Ox (Ox/Ox (10); n = 12) four days after sensitization. Percent change in withdrawal thresholds are shown in Figure 2. (Bottom) Shown are withdrawal thresholds (mean ± SEM; in grams) for Ox-sensitized and challenged ND4 mice that that did not receive intralabiar injection (Ox/Ox/NT), received saline (Ox/Ox/saline), or c48/80 (Ox/Ox/c48-80) on days 5-8 after the last Ox challenge. Percent change in withdrawal thresholds are shown in Figure 5G and Figure S2.

|  |  |  |  | |  | |
| --- | --- | --- | --- | --- | --- | --- |
|  |  | **Day after 10 Challenges** | | | | |
| **Treatment** | **Baseline** | **1** | | **21** | | **42** |
| **NT** | 0.86 ± 0.11 | 0.63 ± 0.06 | | 0.70 ± 0.05 | | 0.72 ± 0.08 |
| **Ox/EtOH (10)** | 0.72 ± 0.06 | 0.54 ± 0.05 | | 0.57 ± 0.05 | | 0.68 ± 0.04 |
| **Ox/Ox (10)** | 0.75 ± 0.10 | 0.22 ± 0.06 | | 0.35 ± 0.03 | | 0.63 ± 0.04 |
| **Treatment** | **Baseline** | **9** | | **21** | | **35** |
| **Ox/Ox/NT** | 0.84 ± 0.05 | 0.36 ± 0.05 | | 0.36 ± 0.04 | | 0.43 ± 0.05 |
| **Ox/Ox/saline** | 0.83 ± 0.11 | 0.40 ± 0.09 | | 0.26 ± 0.02 | | 0.50 ± 0.06 |
| **Ox/Ox/c48-80** | 0.66 ± 0.05 | 0.44 ± 0.10 | | 0.43 ± 0.05 | | 0.46 ± 0.06 |
