# Supplementary material for: Repeated hapten exposure induces persistent tactile sensitivity in mice modeling localized provoked vulvodynia
Source: PLoS One. 2017 Feb 3;12(2):e0169672. doi: 10.1371/journal.pone.0169672 (PMC5291437; doi:10.1371/journal.pone.0169672)
Supplement: S2 Table — (DOCX) [file pone.0169672.s005.docx]

**Table S2.** Lineage and activation markers used to identify skin-infiltrating cells in oxazolone-challenged labia

| **Antigen** | **Color** | **Clone** | **Host** | **Stable Public Identifier** | **Vendor** |
| --- | --- | --- | --- | --- | --- |
| CD3ε | BV650 | 145-2C11 | Armenian Hamster | BD Biosciences cat no. 564378 | BD Biosciences |
| CD44 | BV711 | IM7 | Rat | BD Biosciences cat no. 563971 | BD Biosciences |
| CD4 | APC-eFluor780 | RM4-5 | Rat | AB_1272183 | eBioscience |
| CD11b | eFluor450 | M1/70 | Rat | AB_1582237 | eBioscience |
| CD11c | eFluor450 | N418 | Armenian Hamster | AB_1548654 | eBioscience |
| CD25 | PE | PC61.5 | Rat | AB_465607 | eBioscience |
| CD45R | eFluor450 | RA3-6B2 | Rat | AB_1548761 | eBioscience |
| CD45RB | FITC | C363.16A | Rat | AB_465064 | eBioscience |
| PD-1 | PE-Cy7 | J43 | Armenian Hamster | AB_10853805 | eBioscience |
| CD103 | APC | 2E7 | Armenian Hamster | AB_1106992 | eBioscience |
| CD8α | BV785 | 53-6.7 | Rat | AB_2562610 | BioLegend |
| FoxP3 | FITC | FJK-16s | Rat | AB_465243 | eBioscience |
| IFN-γ | PE | XMG1.2 | Rat | AB_466193 | eBioscience |
