# Supplementary material for: Repeated hapten exposure induces persistent tactile sensitivity in mice modeling localized provoked vulvodynia
Source: PLoS One. 2017 Feb 3;12(2):e0169672. doi: 10.1371/journal.pone.0169672 (PMC5291437; doi:10.1371/journal.pone.0169672)
Supplement: S3 Table — (DOCX) [file pone.0169672.s006.docx]

**Table S3.** Accession numbers for Taqman primer-probe sets (Life Technologies, Carlsbad, CA) for semi-quantitative PCR used to measure relative transcript abundance in Ox-challenged labia

| **Transcript** | **Genbank Accession number** |
| --- | --- |
| β-2-microglobulin (β2m) | Mm00437762_m1 |
| Nerve growth factor (NGF) | Mm00443039_m1 |
| Cell adhesion molecule 1 (CADM1) | Mm00457556_m1 |
| Interleukin-13 (IL-13) | Mm00434204_m1 |
| Interleukin-6 (IL-6) | Mm00446190_m1 |
| Interferon-γ (IFN-γ) | Mm01168134_m1 |
| T-box transcription factor (TBX21) | Mm00450960_m1 |
| Tumor necrosis factor alpha (TNF-α) | Mm00443260_g1 |
| Chemokine C-X-C motif ligand 2 (CXCL-2) | Mm00436450_m1 |
