# Supplementary material for: Repeated hapten exposure induces persistent tactile sensitivity in mice modeling localized provoked vulvodynia
Source: PLoS One. 2017 Feb 3;12(2):e0169672. doi: 10.1371/journal.pone.0169672 (PMC5291437; doi:10.1371/journal.pone.0169672)
Supplement: S4 Table — (DOCX) [file pone.0169672.s007.docx]

**Table S4. Hindpaw withdrawal thresholds to pressure in mice challenged on the labia, and labiar withdrawal thresholds at different stages of the estrus cycle after Ox challenge cessation.** (Top) Hindpaw withdrawal thresholds (mean ± SEM; in grams) for Ox-sensitized ND4 mice challenged on the labia 10 times with either vehicle (Ox/EtOH (10); n = 7) or Ox (Ox/Ox (10); n = 8). Percent change in withdrawal thresholds are shown in Figure S1D. (Bottom) Labiar withdrawal thresholds for Ox-sensitized and challenged mice in different stages of the estrus cycle at day 1 and day 21 post challenge cessation (mean ± SEM; in grams; n = 5-6/estrus stage/time point).

|  |  | **Day after 10 Challenges** | | |
| --- | --- | --- | --- | --- |
| **Treatment** | **Baseline** | **1** | **17** | **21** |
| Ox/EtOH (10) | 5.34 ± 0.38 | 4.25 ± 0.26 | 4.04 ± 0.37 | 4.35 ± 0.62 |
| Ox/Ox (10) | 5.22 ± 0.25 | 4.97 ± 0.30 | 4.30 ± 0.34 | 3.96 ± 0.19 |

|  |  | **Day after 10 Challenges** | |
| --- | --- | --- | --- |
| **Estrus Stage** | **Baseline** | **Day 1** | **Day 21** |
| Proestrus | 0.67 ± 0.04 | 0.25 ± 0.05 | 0.25 ± 0.04 |
| Estrus | 0.67 ± 0.04 | 0.23 ± 0.04 | 0.33 ± 0.06 |
| Metestrus | 0.67 ± 0.04 | 0.26 ± 0.03 | 0.21 ± 0.05 |
| Diestrus | 0.67 ± 0.04 | 0.20 ± 0.02 | 0.23 ± 0.04 |
